# Evaluating a grant development public involvement funding scheme: a qualitative document analysis

**DOI:** 10.1186/s40900-024-00588-w

**Published:** 2024-06-10

**Authors:** Alexis Foster, Sharon Caunt, Holly Schofield, Karen Glerum–Brooks, Samina Begum, Phil Gleeson, Graham Prestwich, Wendy Baird

**Affiliations:** 1 University of Sheffield, Sheffield, Yorkshire, UK; 2 Sheffield Teaching Hospitals Foundation Trust, Sheffield, Yorkshire, UK; 3 University of Leeds, Leeds, Yorkshire, UK; 4 University of York, York, Yorkshire UK; 5 Public Contributor, Sheffield, UK

**Keywords:** Grant development, Public contributors, PPI resources, Early career researchers

## Abstract

**Background:**

Undertaking Patient and Public Involvement (PPI) when developing health and social care research grant applications is critical. However, researchers may not have any funding to undertake PPI when developing grants. In response, the National Institute for Health and Care Research- Research Design Service for Yorkshire and the Humber in the United Kingdom, provided Public Involvement Fund Awards of up to £600 to fund PPI activity when researchers were developing grant applications. Researchers provided post-activity reports about how they utilised the Public Involvement Fund. These reports were analysed with the aim of evaluating the usefulness of the Public Involvement Fund and to provide learning about supporting researchers to undertake PPI when developing grants.

**Methods:**

The project was a qualitative document analysis of 55 reports. Initially a researcher coded four reports and three Public Contributors provided feedback. Researchers coded the remaining reports and identified key findings. A workshop was held with the three Public Contributors to develop the findings.

**Results:**

Researchers accessing the Public Involvement Fund award were generally early career researchers or clinicians who did not have other sources of funding for pre-grant PPI input. Researchers felt the award was useful in enabling them to conduct PPI, which strengthened their grant applications. Some researchers found that the award limit of £600 and guidance encouraging expenditure within three months, made it difficult to undertake PPI throughout the full grant development process. Instead, the majority of researchers consulted Public Contributors on one or two occasions. Researchers struggled to recruit diverse members or run group sessions due to the time pressures of grant deadlines. Researchers wanted training on undertaking PPI alongside the financial support.

**Conclusions:**

Researchers, especially early career researchers found having a Public Involvement Fund award instrumental in enabling them to undertake PPI when developing grant applications. It would be beneficial for similar schemes to be widely available. Schemes need to provide sufficient funding to enable meaningful PPI and allow researchers to hold the award for long enough to facilitate involvement during the whole grant development process. Researchers continue to need training on undertaking PPI.

**Supplementary Information:**

The online version contains supplementary material available at 10.1186/s40900-024-00588-w.

## Abstract (plain language summary)

Undertaking Patient and Public Involvement (PPI) when developing health and social care research grant applications is important. This ensures that patients have a voice in deciding what topics are researched. However, researchers often do not have funding to undertake PPI when developing grants. In response, a regional research advice service in the United Kingdom established a small grant scheme (up to £600) to fund PPI activity. This was called the Public Involvement Fund (PIF). Researchers developing health and social care grant applications could apply. After spending the funding, researchers wrote reports to explain how they used the Public Involvement Fund and the challenges they faced. We analysed 55 reports submitted over a three-year period to understand researchers’ experiences of the fund. Researchers found the funding critical in enabling them to undertake PPI. Many felt their grants were improved from consulting Public Contributors. For example, helping them to decide a topic, changing their research method or choosing a questionnaire. However, researchers sometimes struggled to recruit Public Contributors, particularly when the research was not about a specific health condition. Researchers wanted to be able to have the award for long enough to enable them to involve Public Contributors throughout the whole grant development process. Alongside funding, researchers also need specific training about undertaking PPI when developing grants. For example, how to recruit representative Public Contributors quickly. It is recommended that similar schemes to the PIF are available to enable researchers to fund PPI activities when developing grant applications.

**Supplementary Information:**

The online version contains supplementary material available at 10.1186/s40900-024-00588-w.

## Background

Patient and Public Involvement (PPI) is viewed as essential in health and social care research throughout the whole research cycle including during the development of research grants, delivery of studies, and when disseminating findings [1]. Increasingly health and social care research funders expect researchers to undertake PPI as part of their grant development process and applications may not be successful without involvement [2]. This includes involving Public Contributors in deciding the research topic, designing the research methods, creating the PPI strategy, and co-writing the application [3, 4]. It has been argued that PPI can have a significant impact at the grant development stage in ensuring that the topic is relevant to patients/members of the public, the design and methods are acceptable, and that the PPI strategy for the study ensures meaningful involvement [5].

Despite the importance of PPI when developing grants [3], involvement is often limited and considered tokenistic [6]. Public Contributors often feel their involvement is to ‘rubber stamp’ applications, with researchers rarely involving them meaningfully throughout the whole grant development process [6]. A key reason for the limited involvement is often because researchers do not have funding to undertake PPI when developing grants [7]. In response, the National Institute for Health and Social Care Research- Research Design Services (NIHR-RDS) in the United Kingdom, ran small grant schemes to support researchers with undertaking PPI activity during the grant development process. The NIHR-RDS were regional services, which advised researchers on developing health and social care research grant applications (Research Design Service | NIHR). One PPI scheme was run by NIHR-RDS for the Yorkshire and Humber region (RDS-YH). RDS-YH was one of the first regions to provide researchers with a grant of up to £500 to fund PPI activity during the grant development process. This was called the Public Involvement Fund (PIF) (RDS YH Public Involvement Fund – Research Design Service Yorkshire and Humber (nihr.ac.uk)). Subsequently, all 10 RDS regions in England agreed to establish PPI grants for researchers developing grant applications, using shared principles and increased the value of the award to £600.

As part of the application process, researchers were asked to describe what PPI activities they wanted to undertake during grant development, how they would recruit Public Contributors with lived experience, and how they would evaluate the PPI activity. They were also required to specify the research grant scheme and deadline they were aiming for. Recipients were expected to provide remuneration to Public Contributors at recommended rates e.g. Payment guidance for researchers and professionals | NIHR and were signposted to resources on conducting PPI. For example, Capobianco et al. [8] funded some of their grant development PPI activity through such a scheme.

A condition of the PIF award was for recipients to submit a report of the PPI activities conducted. The reports followed a prescribed template informed by Public Contributors. Recipients were asked to provide a description of the PPI activity undertaken, how input from the Public Contributors influenced the proposed grant and any feedback received from the Public Contributors about how the activity was run. These PIF reports provided a rich data source to understand more about how the PIF supported researchers to undertake PPI during the grant development process and the challenges they faced.

Baxter et al. [7] published an article on the PPI activities undertaken by previous recipients of the PIF. They analysed 25 of the reports returned before 2015. Baxter et al. [7] reported that recipients felt that the PPI input enhanced their grant applications. However, researchers faced barriers to involvement including struggling to recruit Public Contributors and finding it difficult to facilitate PPI groups. Since Baxter et al’s [7] evaluation, PPI practice has evolved including conducting PPI activity online, an increased focus on Equality, Diversity and Inclusion (EDI) and greater expectations from funders that researchers should undertake PPI prior to submitting grant applications. More generally, it has been recommended that further research on undertaking PPI during the grant development process is needed [6]. Given these developments, it is important to evaluate more recent PIF reports with the aim of understanding the impact of the PIF for researchers and their grant applications and the challenges researchers encountered. This learning will support the delivery of future Public Involvement schemes, which will improve PPI during the grant development process in the long-term.

## Methods

We used qualitative document analysis [9, 10] to explore the reports returned by researchers who had received a PIF award from NIHR RDS for Yorkshire and Humber. We involved Public Contributors and utilised the GRIPP2 Checklist (Supplementary File 1).

There were 55 reports returned between 2019 and 2022. PIF recipients were either academic researchers or health/social care/public health professionals developing research grant applications. Within this article, we refer to everyone as ‘researchers’. Researchers were given an outline structure for their reports (as described in the background and Supplementary File 2). Each report contained differing levels of detail. For example, some researchers described the demographics of Public Contributors whereas other researchers did not include this information. All of the reports were submitted by researchers based in the Humber and Yorkshire region of the United Kingdom. However, researchers could undertake the PPI activity in any geographic region.

Qualitative document analysis seeks to develop learning through identifying recurring patterns, known as themes within the text [9]. We drew upon the principles of thematic analysis to help us identify themes arising from the reports [11]. Initially, a researcher (AF) undertook familiarisation and initial coding through reading four of the reports. Coding involved identifying arising topics within the different reports to create a list of issues that we wanted to explore across the reports. This is called a ‘coding framework’. These four reports were selected from different years and were longer reports to provide more information to inform the initial coding framework developed by AF. At that stage, three Public Contributors also read the four reports, reviewed the initial coding, and provided feedback to AF. For example, the Public Contributors said it was important to reflect on what was not said within the reports and the skill gaps/training needs of researchers. AF used the feedback to develop the coding framework.

To undertake document analysis of the 55 reports, four researchers (AF, SC, HS, KGB) undertook thematic analysis on the 55 reports. The analysis was undertaken between November 2022 and March 2023. As the analysis process involved Public Contributors and less experienced qualitative researchers, the analysis was undertaken using Microsoft Word rather than a specific qualitative analysis software. Coding was undertaken using the comment function on Word with AF then collating the comments into a new document.

Each report was analysed by one researcher. Initially, the researchers familiarised themselves with a report by reading it several times. Researchers then coded the report using the coding framework. Where a researcher came across information that did not fit into an existing code, they emailed the research team to agree a new code. AF then updated the coding framework. Once all the reports were coded, AF collated the codes. By grouping the codes by commonality, AF was able to develop initial themes. In discussion with the other researchers, AF reviewed, defined, and named the themes. AF generated an initial summary report capturing the emerging analysis.

Alongside the thematic analysis, we used numerical counting of information when relevant. For example, counting how many researchers used specific types of recruitment methods.

After producing the initial summary report, a workshop was held with the three Public Contributors. The three-hour workshop was held virtually in March 2023. The Public Contributors were sent the initial report in advance of the workshop. AF presented the emerging findings and the Public Contributors provided feedback on how the themes could be further developed and the implications of the findings. For example, the Public Contributors wanted greater reflection on how the grant recipients continued to involve people after they spent the PIF. The Public Contributors’ feedback was used to support the development of the findings report. This manuscript is based on the findings report. AF led the writing of the article, with Public Contributors reviewing and providing input into the drafts and revised manuscript. The Public Contributors are co-authors.

Ethical approval was not required. This was because the study involved analysing reports that were secondary data sources. No primary data was collected. As part of accepting the terms and conditions of the PIF, researchers consented to their reports being analysed by the RDS-YH for performance and evaluation purposes. The consent included utilising de-identified content from the reports as quotes. No personal identifiable data from the reports was shared with the Public Contributors. For example, researcher name and institution were redacted before the reports were sent to them.

## Findings

The PIF appeared instrumental in enabling recipients, especially early career researchers to undertake PPI when developing grants. Researchers faced challenges when undertaking PPI which arose from both the structure of the PIF and the specific context of grant development. We explore these issues through six themes:

1. Characteristics of the PIF recipients and the proposed research.
2. Impact of the PIF.
3. PPI activity funded through the PIF.
4. The structure of the PIF constraining involvement.
5. Challenges recruiting diverse PPI members.
6. Need for PPI training.

### Characteristics of the PIF recipients and their proposed research

One hundred and seven PIF awards were granted between April 2019 and November 2022. At the time of the analysis, 68 of these awards had been claimed following the completion of PPI activities. Fifty-five researchers returned reports. The reports were based on 80.8% of PIF awards claimed. Some researchers had not returned reports because the PPI activities were still ongoing. However, other researchers did not claim the PIF funding because they were no longer applying for the grant due to work/personal circumstances. For example, researchers who moved to a clinical role or researchers who decided not to pursue their research idea. Other researchers did not claim the PIF because their PPI activity incurred minimal costs. For example, attendees may have chosen not to receive payment or in-person meetings were changed to online meetings, which did not incur travel/subsistence costs.

There was considerable diversity in the healthcare topics that researchers focused on, and this had implications for the challenges researchers faced when undertaking PPI. Topics included the acceptability of a pain relief intervention during childbirth, mental health peer support workers, and the use of video appointments for people with cystic fibrosis.

Generally, PIF recipients were early career researchers. Examples included clinicians applying for doctoral fellowships or researchers developing their first grant as a lead applicant.

### Impact of the PIF

Researchers welcomed the PIF because it enabled them to undertake PPI activity that enhanced their grant applications. Researchers provided examples of how their ideas had developed following PPI input funded by the PIF. This included refining the research question, choosing outcome measures, identifying participant recruitment methods, and designing the future PPI plan. Many researchers reflected that without the PIF they would not have been able to consult Public Contributors or would have relied on people giving their time without remuneration. Some researchers credited the PIF as making the difference of their grant application being successful.

“*We firmly believe that Research for Patient Benefit should include the expertise of patients, and the fund allowed us to include patient’s expertise in the design of our project.*” [P1].

### PPI activity funded by the PIF

The majority of the researchers used the PIF to consult Public Contributors once or twice during a specific part of the grant development process. Specific development stages included [1] Identifying and prioritising the topic [2], Designing the study and [3] Development of the grant application itself.

Several researchers used the PIF to identify the specific focus of the research proposal. For example, one researcher asked Public Contributors about what research is needed on the role of pharmacists within general practices. Another researcher worked with women who had experienced a rare pregnancy complication to identify what research they felt was needed on the topic.

Some researchers sought PPI input into designing the study including deciding the methods, participant recruitment processes and reducing participant burden. For example, one researcher consulted Public Contributors on which outcome measures to use and another sought advice on when a proposed questionnaire should be administered. A small number of researchers used the PIF to fund PPI input at the latter stages of the grant development process. For example, asking Public Contributors to review the Plain English Summary of their grant application form.

Only a small number of researchers used the PIF to support PPI input through the whole grant development process. For example, one researcher over a three-month period created a PPI group of 5 Public Contributors. They were consulted several times to identify the topic, design the study, and review the Plain English Summary. The researcher was able to involve Public Contributors throughout the whole grant application process because all of the engagement was virtual, and the researcher had the capacity to develop and submit their grant application within three months.

*“Mutually convenient meetings, each of 1–2-hour duration were arranged for all 5 PPI members and 5 virtual meetings were held”.* [P4]

Researchers sought to hold group consultations but some had to take a pragmatic approach due to grant deadlines or challenges recruiting PPI members. Most researchers held group consultations. Some researchers ran repeated meetings with the same Public Contributors, whereas others had different people attending each consultation to widen involvement. However, sometimes researchers held an individual meeting with a Public Contributor, had email exchanges or utilised questionnaires. This was due to researchers being unable to get all Public Contributors together at the same time or Public Contributors not feeling able to participate in group meetings. There was acknowledgement from some researchers that there was a loss of collective contributions from not having group consultations but that a pragmatic approach to involvement was necessary especially when there were time pressures such as an impending grant deadline.

*“Two online discussion groups were organised, some people stated they wished to be involved but did not like discussion groups or could not make the events, these people completed questionnaires instead.”* [P2].

Since COVID-19, most researchers held activities online albeit with a few exceptions. The exceptions were generally when researchers felt that the population group would struggle to engage online. For example, when the target population spoke English as a second language. Researchers felt online engagement facilitated involvement. This included being able to involve people from a wider geographical area and people with work or child/carer commitments. Researchers reflected in the reports how online groups were cheaper to run because there was no travel, refreshment or room hire costs. Researchers reported that this enabled them to use the PIF to involve more people or to consult Public Contributors a greater number of times.

“*As the majority of those who expressed interest preferred to have a virtual meeting or were happy with either virtual or in-person, with the RDS’s agreement we repurposed the budget that we put aside for travel and subsistence fees and recruited a 7th person to the Patient Advisory Group.”* [P1].

Some of the researchers described how they had planned to continue to keep the Public Contributors involved. This was usually through involving one or two of the Public Contributors as named PPI Co-Applicants on the grant application or by inviting the Public Contributors to form part of an advisory panel if the grant was successful.

### The structure of the PIF constraining involvement

Researchers were constrained in undertaking extensive PPI because of the PIF limit of £600. Researchers had to balance the number of Public Contributors they involved with the number of times they consulted them. The most common type of involvement was researchers running two consultations with different people attending each. For example, one researcher ran two consultation groups, with four Public Contributors attending the first meeting and three different people attending the second. This type of involvement meant that PPI members were generally consulted only on specific elements of the grant proposal rather than being active partners engaged in co-designing the whole project. Researchers reported that when they used the PIF to help them decide the research topic and initial design, they then had no other sources of funding to resource further PPI as they developed their grant application.

Researchers also felt constrained that they were encouraged to spend the PIF within three months and often requested to have the award for longer. Only 12 PIF awards were completed within the three-month time frame. The average award length was 6.6 months, but ranged from 2 to 21 months. Researchers, especially early career researchers reported that it could take them at least a year or longer to develop their research from deciding the topic to submitting a grant application and ideally wanted the PIF throughout the period to enable them to undertake involvement throughout the grant development cycle.

### Challenges recruiting diverse PPI members

Some researchers faced challenges recruiting Public Contributors and had to try alternative recruitment methods after struggling to identify people through their initial attempts. This was challenging because of the time pressures associated with developing grants. The most common recruitment method was through healthcare services (*n* = 23) (Table 1). Other common methods included through social media e.g., Facebook peer support groups (*n* = 17), charities (*n* = 14) and attending an existing PPI group (*n* = 11). Only two researchers used the People in Research database. This is a database in the UK that links researchers with Public Contributors (Home - People in Research). Other recruitment methods included through Community Leaders and consulting Public Contributors involved in existing studies. The success of different approaches was dependent on the specific topic and whether a researcher had prior links with the target population. For example, clinical researchers within a healthcare service were able to successfully recruit Public Contributors with relevant lived experience, but researchers without a prior relationship found it difficult/were unable to recruit the target population. Researchers found it more challenging to recruit people when developing methodological or public health research applications compared to when the research was focused on a specific health condition.

*“It was not possible to recruit from sources outside of the families I could access directly through my own service. This will be important to consider when designing recruitment strategies for the research itself.”* [P3].

**Table 1:** Recruitment methods of PPI members

Recruitment method	Number of researchers (*n* = 93)
NHS Clinical services	23 (24.7%)
Social media/Facebook group	17 (18.3%)
Charity	14 (15.1%)
Existing involvement group	11 (11.8%)
Advertisement	7 (7.6%)
Community groups/support groups	6 (6.5%)
Did not say	3 (3.2%)
Existing study	2 (2.2%)
Via a trusted community member	2 (2.2%)
People in Research database	2 (2.2%)
Existing contacts	2 (2.2%)
Posters	2 (2.2%)
Through other clinical researchers	1 (1.1%)
Existing contacts	1 (1.1%)

Total more than 55 because researchers often used multiple methods of recruitment

Some researchers had to try different recruitment methods because of struggling to recruit people initially. This required additional time and effort, which was challenging when researchers had a grant deadline.

“*The main difficulty we encountered was the short time frame in which to organise this focus group due to the grant submission deadline. This meant that we were a little limited in the number of patients we were able to recruit.”* [P5].

After struggling to recruit Public Contributors through other methods, researchers often resorted to accessing existing PPI groups. Researchers foundthis approach both helpful and challenging. They reported an advantage of consulting existing PPI groups was that members were engaged and experienced in research. However, researchers reported that members did not always have specific experience with the condition or service that the grant application was focused on. Furthermore, researchers were often limited to one-off, short discussions with existing groups which some researchers felt was tokenistic.

Researchers experienced challenges involving diverse Public Contributors because of time and opportunity constraints. By diverse Public Contributors, we mean people with different demographics including ethnicities, ages and socioeconomic status. Researchers reported that they had to involve Public Contributors that were readily accessible to them because they did not have time to invest recruiting diverse members due to impending grant deadlines. For example, one researcher approached patients attending a specific service, but this was based in a town which had a predominantly White British population. In other cases, researchers struggled to identify Public Contributors to participate so had to involve who they could rather than being able to select a diverse range of Public Contributors.

**“***The main difficulty we encountered was the short time frame in which to organise this focus group due to the grant submission deadline. This meant that we were a little limited in who we were able to recruit.*” [P6].

### Need for training on undertaking PPI alongside financial support

Some of the PIF recipients found conducting PPI challenging because of their lack of PPI experience. This was especially in terms of undertaking rapid recruitment of Public Contributors and facilitating groups such as managing dynamics and planning session content. Researchers felt that practical training and opportunities to observe other researchers undertaking PPI activities would help them to develop their skills.

## Discussion

The PIF was essential in enabling predominately early career researchers to undertake PPI when developing grant applications. However, the structure of the PIF and impending grant deadlines meant that researchers undertook fairly limited PPI activities that were of a consultative nature and often did not involve diverse Public Contributors and therefore perspectives. Alongside funding, researchers also would have benefited from training on conducting PPI.

The availability of the PIF award filled an important funding gap. This was especially the case for early career researchers. This may be because more established researchers have existing budgets they can utilise or have established PPI groups that they can consult. Our finding that the PIF enabled PPI activity reflects other literature. Aas et al. [12] highlighted that researchers found it difficult to access funding for PPI activity during the grant development process. Previous work by Boote et al. [13] quantified how investment of £1 in a PPI grant resulted in £395 of grant capture. Thus, PPI development grants can have a tangible impact on generating research funding. As health, public health, social care and life sciences research funders increasingly want Public Contributors to be involved with developing grant applications then there is a need for someone, be it funders themselves, higher education institutions or research development organisations to resource involvement during the grant development process.

However, the specific nature of the RDS-YH-PIF award in terms of providing a maximum of £600 and asking researchers to try and spend the money within three-months, meant that some researchers struggled to undertake PPI activity throughout the grant development process. Whilst the PIF was not established with the intention of necessarily funding involvement through the grant development phase, the absence of other sources of funding means that schemes which fund meaningful involvement throughout the whole grant development process are needed. Ní Shé et al. and Ocloo et al. [6, 14] have reported that PPI during the grant development stage is often tokenistic and it is important the Public Involvement funding schemes are structured to facilitate inclusive involvement throughout the process.

Researchers had to claim the PIF money after running activities because of host institutional financial processes. Whilst none of the recipients reported that this was a barrier, future schemes may want to consider alternative processes in cases where this could be problematic. For example, the funding provider booking travel for Public Contributors on behalf of the researchers. It is also important to acknowledge the need for funders to have appropriate financial structures to enable researchers to hold PPI grants for longer periods.

Public Contributors were recruited via different methods and the success of the approach was dependent on the topic area. However, researchers often struggled with recruitment, and it often took longer than anticipated. It appeared that it was more challenging to recruit Public Contributors to support public health or methodological studies. This was because researchers could utilise healthcare services, topic specific charities, and social media peer groups for specific health condition topics. These opportunities were not available for other topic areas.

Struggling with recruitment was particularly problematic when researchers had impending grant deadlines. Consequently, researchers may want to utilise several recruitment methods simultaneously to maximise their chances of recruitment and utilise methods that have previously been successful for the population group. Our findings reflect previous studies including Baxter et al. and Gilfoyle et al. [7, 15] about the challenges of recruiting Public Contributors and greater nuanced learning of effective methods for different topic areas. Only a small number of researchers recruited Public Contributors through the People in Research database (Home - People in Research). There is significant scope for researchers in the United Kingdom to make better use of this resource.

Despite recognising the importance of involving Public Contributors, few researchers actively sought to recruit a diverse group of Public Contributors. Researchers reflected that due to recruitment challenges or time pressures, their priority was having people with lived experience involved. Researchers often involved the first people willing to volunteer rather than spending time to proactively seek Public Contributors that represent the diversity of the target population. This reflects Ocloo’s et al. [14] systematic review of reviews that ensuring the diversity of Public Contributors was problematic and the practices of researchers could exacerbate inequalities. For example, some researchers recruited via community leaders but did not renumerate them for their support. Our evaluation in combination with the increased focus on diversifying PPI activity over recent years [16] has highlighted the need for improvement in PPI during the grant development process. In addition, to facilitate the extra time and resources required to increase the diversity of Public Contributors, research funders also need to adapt their processes.

Researchers utilised pragmatic approaches to involvement as it was not always feasible to conduct group consultations. These experiences highlight the need for researchers to be aware of, and consider different ways of undertaking PPI to maximise involvement. This reflects other literature on PPI that recognises involvement can take different forms including questionnaires, individual consultations and email exchanges, and the need for researchers to evolve from proposing group consultations as standard [17].

Researchers need training on undertaking PPI, including having opportunities to observe others facilitating PPI. There is an expectation that researchers know how to conduct PPI, but it is a skill that researchers require training in [18]. However, to date there is a gap in the literature on delivering training on undertaking PPI during the grant development phase.

### Implications for policy and practice

Having schemes like the RDS-YH PIF are essential to enable researchers to undertake PPI when developing grant applications. Research funders and institutions need to provide funding schemes to enable researchers, especially early career researchers to undertake meaningful PPI throughout the grant development process. For example, funders could offer pre-award PPI grants. We have identified a number of recommendations for such schemes (Table 2).

Public involvement funding schemes need to provide funding of about £1000 (or equivalent) that can be spent over at least a year period to enable researchers to involve Public Contributors throughout all stages of the grant development process. Funders and host organisations need to explore methods of supporting researchers who cannot afford to claim the funding retrospectively.

Researchers need to consider the most fruitful recruitment methods for their specific topic and utilise a number of recruitment methods from the start to maximise interest. If researchers are recruiting via community leaders/voluntary sector organisations, they also need renumerating.

Researchers need to be pragmatic about the involvement methods they use. Alternative involvement methods other than group consultations may be more feasible. Encouraging virtual rather than face-to-face meetings enables involvement budgets to be stretched further, but the risk of excluding people who cannot or would prefer not to engage online must be considered.

Researchers need training on undertaking PPI throughout the grant development process. This includes on recruiting diverse Public Contributors rapidly, different involvement methods, and facilitation skills.

This paper is based on one PPI funding scheme. Further research comparing different schemes is needed to enable further learning on how best to fund PPI during the grant development process.

**Table 2:** Recommendations for public involvement funding schemes

Recommendation	Details
Availability of grant development public involvement funding schemes which are of sufficient value and duration	Need for research organisations to provide funding to enable researchers to undertake PPI during grant development. Schemes need to be of sufficient value and length to enable researchers to undertake meaningful PPI throughout the grant development process.
Knowledge of appropriate recruitment methods for the population group/topic and use of multiple recruitment methods ensuring diverse Public Contributors.	Researchers need to recruit through a number of methods that are appropriate for their topic area including seeking diverse Public Contributors.
Pragmatic approach to involvement	Researchers may want to consider other involvement methods rather than group meetings including email discussions, individual consultations, and surveys.
Virtual rather than in-person	Consideration of holding meetings online rather than in person (if appropriate and with appropriate support for Public Contributors) to enable researchers to maximise involvement within funding constraints.
Training on undertaking PPI during the grant development process	Researchers need training on undertaking PPI during the grant development process including on rapid recruitment and group facilitation skills.

### Strengths and limitations of the research

A key strength of the research is that it provides valuable learning on how to support researchers to undertake PPI during the grant development process. The study benefited from involving Public Contributors in supporting the analysis and considering the implications of the findings. However, the research was based on analysing reports provided by recipients of the NIHR-RDS-YH PIF award, and it is unknown whether the learning reflects experiences of similar schemes. Our findings were compatible with the wider literature, indicating that the findings are relevant beyond this specific context.

### Conclusion

PPI funding schemes are essential for facilitating Public Contributor input during the grant development process. Funding schemes need to be of sufficient monetary value and be awarded for over a year period to enable researchers to undertake PPI throughout the grant development process. Researchers also need PPI training alongside financial support to maximise the impact of PPI activity.

### Electronic supplementary material

Below is the link to the electronic supplementary material.

Supplementary Material 1

Supplementary Material 2

## Data Availability

Data sharing is not applicable to this article as no datasets were generated or analysed during the current study.

## Abbreviations

EDI: Equality, Diversity and Inclusion
GRIPP: Guidance for Reporting Involvement of Patients and the Public
NHS: National Health Service
NIHR: National Institute for Health and Care Research
PIF: Public Involvement Fund
PPI: Patient and Public Involvement
RDS: Research Design Service
RDS-YH: Research Design Service for Yorkshire and Humber
