# Supplementary material for: Evaluating a grant development public involvement funding scheme: a qualitative document analysis
Source: Res Involv Engagem. 2024 Jun 10;10:57. doi: 10.1186/s40900-024-00588-w (PMC11163746; doi:10.1186/s40900-024-00588-w)
Supplement: Supplementary file 1 — Supplementary Material 1 [file 40900_2024_588_MOESM1_ESM.docx]

GRIPP2 reporting checklists [1]

| Section and topic | Item | Reported on page No |
| --- | --- | --- |
| 1: Aim | Report the aim of PPI in the study | 6 |
| 2: Methods | Provide a clear description of the methods used for PPI in the study | 6-8 |
| 3: Study results | Outcomes—Report the results of PPI in the study, including both positive and negative outcomes | 8-16 |
| 4: Discussion and conclusions | Outcomes —Comment on the extent to which PPI influenced the study overall. Describe the positive and negative effects | 16-19 |
| 5: Reflections/critical perspective | Comment critically on the study, reflecting on the things that went well and those that did not, so others can learn from this experience | 19 |

PPI = patient and public involvement

[1] S. Staniszewska *et al.*, ‘GRIPP2 reporting checklists: Tools to improve reporting of patient and public involvement in research’, *BMJ*, vol. 358, 2017, doi: 10.1136/bmj.j3453.
