# Supplementary material for: Evaluating a grant development public involvement funding scheme: a qualitative document analysis
Source: Res Involv Engagem. 2024 Jun 10;10:57. doi: 10.1186/s40900-024-00588-w (PMC11163746; doi:10.1186/s40900-024-00588-w)
Supplement: Supplementary file 2 — Supplementary Material 2 [file 40900_2024_588_MOESM2_ESM.docx]

**Supplementary File 2**

**Structure of the PIF Report**

- Background to the grant application.
- Why you applied for the award, what you wanted patients and the public to do.
- How you went about identifying and recruiting relevant patients and the public to take part, and a description of how you engaged with them (e.g. did they take part in consultation meetings/focus groups, or bid-writing meetings etc.)
- Description of the type of people who took part, and how many people took part in each relevant engagement activity.
- Description of the contributions made by the patients and the public, and a consideration of what was changed or adapted as a result of their involvement.
- A discussion of how you evaluated the involvement of the patients and the public, together with a short account from at least one person you involved wherever possible
- A consideration about how patient and public involvement will be taken forward should you be awarded funding.
- A consideration of any difficulties encountered and if there are things you might have done differently.
- Notification of whether or not you were successfully awarded funding or the date of when you expect to hear confirmation.
- Appendix: detailed breakdown of how the funding was spent.
